# Machine Learning Enabled Graph Analysis of Particulate Composites: Application to Solid-State Battery Cathodes

**DOI:** 10.1021/acsenergylett.5c04258

**Published:** 2026-05-14

**Authors:** Zebin Li, Shimao Deng, Yijin Liu, Jia-Mian Hu

**Affiliations:** † Department of Materials Science and Engineering, 5228 University of Wisconsin-Madison, Madison, Wisconsin 53706, United States; ‡ Walker Department of Mechanical Engineering, 12330 University of Texas at Austin, Austin, Texas 78712, United States

## Abstract

Particulate composites underpin many solid-state chemical and electrochemical systems, where microstructural features such as multiphase boundaries and interparticle connections strongly influence system performance. Advances in X-ray microscopy enable capturing large-scale, multimodal images of these complex microstructures with unprecedentedly high throughput. However, harnessing these data sets to discover new physical insights and guide microstructure optimization remains a major challenge. Here, we develop a machine learning (ML)-enabled framework that enables automated transformation of experimental multimodal X-ray images of multiphase particulate composites into scalable, topology-aware graphs for extracting physical insights and establishing local microstructure–property relationships at both the particle and network level. Using the multiphase particulate cathode of solid-state lithium batteries as an example, our ML-enabled graph analysis corroborates the critical role of triple-phase junctions and concurrent ion/electron conduction channels in realizing desirable local electrochemical activity. Our work establishes graph-based microstructure representation as a powerful paradigm for bridging multimodal experimental imaging and functional understanding and facilitating microstructure-aware data-driven materials design in a broad range of particulate composites.

## Graphical abstract

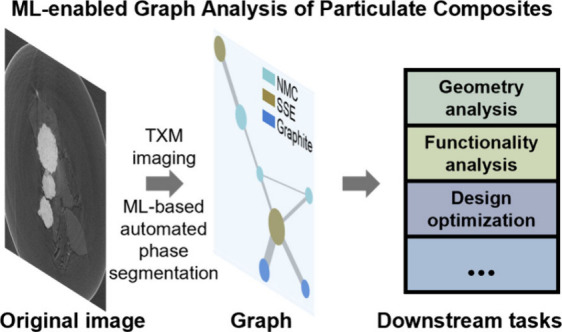

Particulate composites are widely used in solid-state chemical and electrochemical systems, including solid-state batteries (SSBs),^1^ solid oxide fuel cells (SOFCs),^2^ catalysts for fluid catalytic cracking (FCC) in the petroleum industry,^3^ and advanced sensors.^4^ The microstructure of these particulate composites, notably the phase boundaries and interparticle connections, can critically affect their properties and the system performance. For example, in the process of refining petroleum through FCC, the catalytically active sites are the ultimate drivers that chemically convert the feedstock into the targeted chemicals.^3^ In SOFCs, the three-phase (i.e., cathode, electrolyte, and air) boundaries are the active sites for the oxygen reduction reaction.^5^ Similarly, in SSBs, the active materials, ionic conductors, and electrical conductors need to work together seamlessly to enable on-demand energy storage and release.

Although the critical role of the microstructure is well recognized, its impact on the properties of particulate composites remains challenging to quantify. Recently, advances in electron and X-ray microscopy tomography enable the acquisition of large-volume, multimodal images for microstructures of particulate composites.^6^ To connect these high-dimensional microstructure data sets to the properties and performance, and in future, the parameters of synthesis, processing, and manufacturing, it is necessary to obtain a lower-dimensional representation that captures the most salient features of a given microstructure. Specifically, for representing the microstructures of multiphase particulate composites, existing methods, which are based on the image pixels/voxels^7–12^ or statistical correlations,^13–17^ would not suffice for two reasons. First, it would be computationally expensive to apply them to microstructures of practical size that contain billions of voxels. Second, the connectivity and topological characteristics of the particles and other constituent phases, which critically determine the materials properties and system performance, cannot be encoded into the low-dimensional representation via these methods.

To address this challenge, here we use graphs to represent the microstructures of particulate composites, which has three main advantages. First, since the individual particles/phases almost always occupy more than one pixel/voxel in the raw data, graph-based representation would be computationally more efficient than image-based representation and permits incorporating thousands of particles with minimal memory cost.^18^ Second, by representing individual particles/phases as nodes and their interfaces as edges, the connectivity and topology of the particles/phases are naturally retained. Third, by storing both the structural and functional features of individual particles/phases as well as the interfaces into the node and edge feature vectors, respectively, graph would facilitate multiscale, multimodal information fusion and the development of local microstructure–property relationships. Thus far, graphs have been extensively used to represent polycrystalline microstructures, which has in turn enabled the application of graph neural network (GNN) to link the low-dimensional embedding of the polycrystal graph to properties.^18–23^ However, to our knowledge, graphs have not yet been applied to represent the microstructures of multiphase particulate composites. This is in part due to the challenge of achieving an accurate and automated segmentation of the individual phases from the experimentally measured raw microstructure images, which can now thankfully be addressed by leveraging advanced machine learning (ML)-powered computer vision models.^24–29^

As an example, here we demonstrate the use of graphs to represent the experimentally measured microstructure images of the composite cathode of SSBs, which is composed of mixed active particles (LiNi*_*x*_* Mn*_*y*_* Co*_*z*_* O_2_, *x* + *y* + *z* = 1, donated as NMC), solid-state electrolytes (SSEs), and conductive carbon (graphite) that we show can be automatically segmented by ML models. We then discuss how such graph-based representation can be used to facilitate the study of the microstructure–property relationships at both the particle and the network level, and to evaluate the alignment of microstructure design rules. To that end, we first acquire high-resolution, chemically resolved imaging of SSB cathodes using full-field X-ray imaging, and then develop a workflow that capitalizes on ML-based phase segmentation to automatically convert the original X-ray images to graphs. Notably, both the morphological features and the local electrochemical properties of NMC particles (i.e., Ni oxidation state, represented by the Ni K-edge energy^30–32^ ) are incorporated into the feature vectors of the nodes/edges of the graphs. Building on such node-/edge-level correlations, we establish an understanding of the local microstructure–property relationship in such a complex mesoscopic system. Last but not the least, we demonstrate that graph enables a GNN-based property prediction at the node level, representing a critical step for the realization of microstructure-aware inverse design of processing and synthesis parameters that yield the desirable material properties.

The demonstration of the SSB system is shown in Figure 1a, where the cathode is composed of a mixture of NMC particles, SSEs, and graphite. During discharging, Li^+^ and electrons migrate through the networks of SSEs and graphite, respectively, both ultimately reaching the NMC particles for Li intercalation. Such an intercalation reaction would be most efficient at the NMC-SSE-graphite triple phase boundaries (TPBs), where the concentrations of Li^+^ and e^–^ are simultaneously high. An equally important microstructure feature is the concurrent Li^+^/e^–^ channels between NMC particles. For example, NMC–SSE–NMC and NMC–graphite–NMC connections can provide channels for the migration of Li^+^ and e^–^, respectively. To analyze these local microstructure features and their correlations with local properties, we apply energy-resolved transmission X-ray microscopy (TXM), which enables the simultaneous spatial mapping of phase morphology and the electrochemical states of NMC particles, resulting in multimodal imaging (Figure 1b). The zoomed-in figures of Figure 1b highlight the TPBs (top panel) and the concurrent Li^+^/e^–^ channels (bottom panel). The original X-ray image and the corresponding TXM image are shown in Supporting Figure S1. Figure 1c shows the workflow of converting raw X-ray images into graphs and the downstream analyses that relate local geometrical and topological features to local electrochemical states.

**Figure 1:**
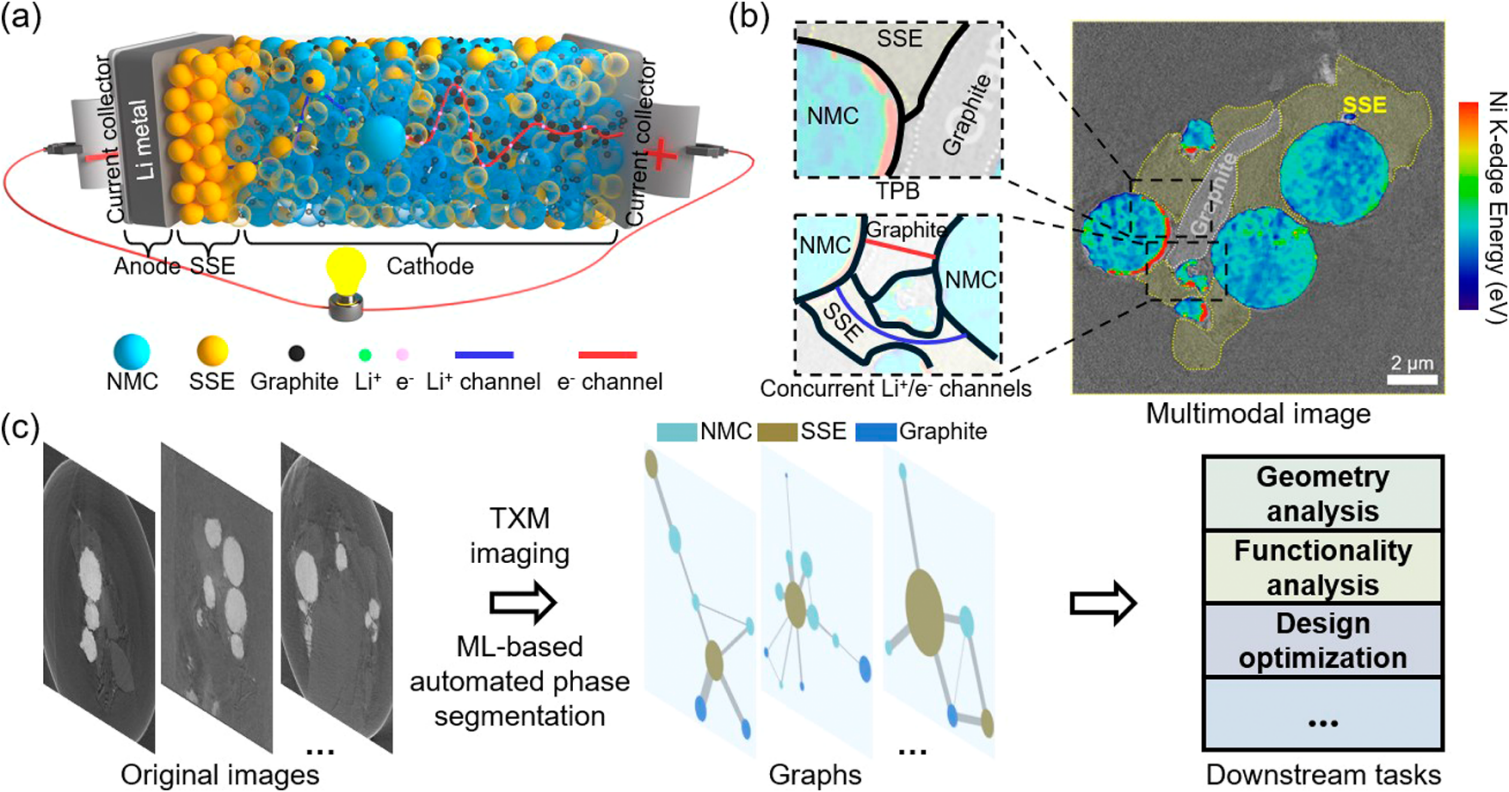
Graph representation and analyses of multiphase particulate composites, using the composite cathode of the solid-state battery (SSB) as an example. (a) Illustration of an SSB. (b) Example of a multimodal image integrated from a 2D X-ray image with human-expert-annotated phases and the corresponding local electrochemical states (i.e., Ni oxidation states, represented by the Ni K-edge energy) of NMC particles obtained by TXM imaging. Zoomed-in figures demonstrate the two critical microstructure features, i.e., triple phase boundaries (TPBs) and concurrent Li^+^/e^–^ channels via the connecting SSE/graphite. (c) The ML-enabled workflow for automatically converting raw X-ray images into microstructure graphs for facilitating and enabling various downstream tasks.

Figure 2 shows an example (from the testing data set) of the original X-ray image of SSB cathodes, the phase segmentation results from a human expert (ground truth) and a customized U-Net (see Supporting Information), and the constructed graph. More examples are shown in Supporting Figure S2. Moreover, based on the instance segmentation map (Supporting Figure S3), we assign identical numbers to each object in the segmented phase map and the corresponding node in the graph. Because its end nodes are explicitly labeled, each edge of a graph, corresponding to the interface of two neighboring objects, can also be uniquely identified. Together, the microstructure graph we constructed allows for compiling physical features for each node and edge and digitalize the neighboring relationships of these individual objects in the form of an adjacency matrix, which are necessary for the application of GNN to link either microstructure graphs^20–23^ or atomic-scale structure graphs^33–37^ to properties.

**Figure 2:**
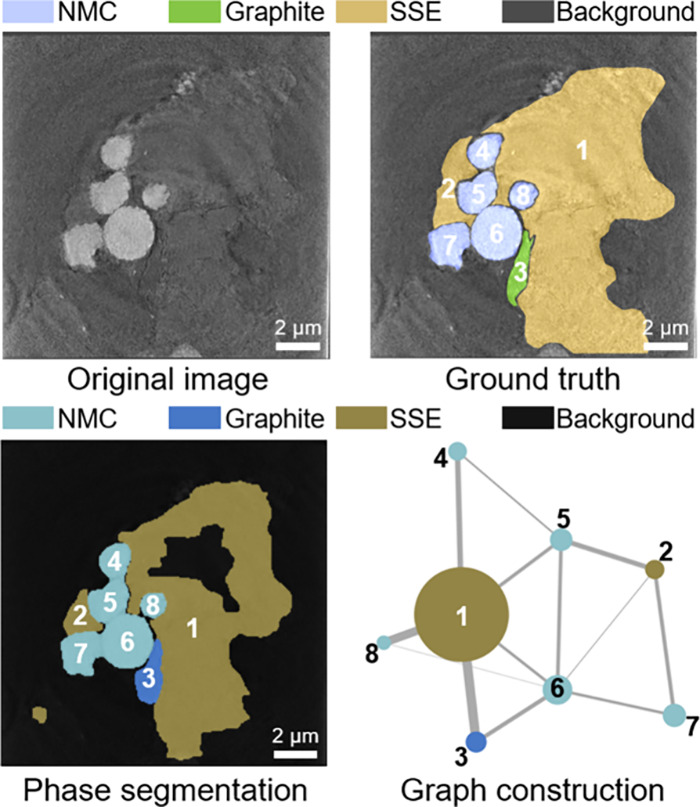
Example results of graph construction enabled by ML-based automated phase segmentation. In the constructed graphs, the node sizes and the edge thicknesses represent the sizes and the interface areas of the corresponding objects and connections, respectively.

Despite the challenges arising due to the similar pixel intensity distributions of monolithic phases in the original X-ray images (Figure S1b), the U-Net-based phase segmentation is largely successful, especially for NMC particles. The automatically constructed graph (bottom right panel of Figure 2) mostly captures the real geometric connections, where the neighboring objects are connected by edges. The edge thickness is proportional to the interface area, which is quantified by the number of overlapping pixels. The node size is proportional to the number of pixels in each object.

Recent works have demonstrated that the design of microstructure morphology is key to achieve efficient charge–discharge at the particle level.^38^ The spatial arrangements of NMC–graphite and NMC–SSE contracts, as well as the resulting TPBs, are pivotal for providing balanced fluxes of Li^+^ and e^–^ (*J* ^Li^ ≈ *J* ^e^) for active cathode materials. Therefore, the optimization of NMC particle size is a nontrivial task. Smaller particles exhibit shorter diffusion length within the NMC particle and are more favorable for fast charging applications. On the other hand, large particles have a higher probability of accommodating multiple TPBs on their surface, integrating the particle into well-configurated conductive networks for both Li^+^ and e^–^. Despite tremendous optimization efforts, the exiting manufacturing method does not have a full control of the electrode micromorphology. Therefore, the high-resolution and large field-of-view microscopic characterization is critically needed to inform the improvement of the manufacturing protocol.

In addition to the experimental characterization efforts, high-throughput quantification of the imaging data is indispensable. Although one can evaluate whether the cathode microstructure of SSBs meets these design targets by directly analyzing the phase-segmented microstructure image, it is often a tedious and labor-intensive process. Instead, performing such an evaluation on microstructure graphs will enable automated and high-throughput statistical analyses. For demonstration, we apply our ML-enabled graph construction to 73 new original X-ray microstructure images, yielding automated construction of 73 microstructure graphs. Figure 3a shows the distributions of TPB size (precisely speaking, the perimeter of TPB) with respect to the ratio of the NMC–SSE interface area to the NMC–graphite interface area, which affects *J* ^Li^/*J* ^e^ by modulating their respective interfacial resistance. An ideal microstructural configuration would offer a large TPB size and a balanced and consistent value of interfacial area ratio, thus stabilized and optimized *J* ^Li^ and *J* ^e^ for every single cathode particle. The data in Figure 3a, however, demonstrate that most of TPBs concentrate in the left part and have ratios that are significantly scattered. Figure 3b shows the distribution of the NMC particle size (pixel count) with respect to the number of TPBs each particle is connected to (only those NMCs associated with TPBs are counted). The data show that most NMC particles are only involved in one or two TPBs, which is less than ideal. Furthermore, it is desirable to establish concurrent Li^+^/e^–^ channels by connecting two NMC particles via both the SSE and graphite (see the bottom inset in Figure 1b). Yet, as shown in Figure 3c, most of NMC particles are only connected to SSE, with limited direct contact with graphite (hence, the *J* ^Li^ and *J* ^e^ are imbalanced). These morphology analyses indicate that there is plenty of room for improving the performance of SSBs through microstructure engineering.

**Figure 3:**
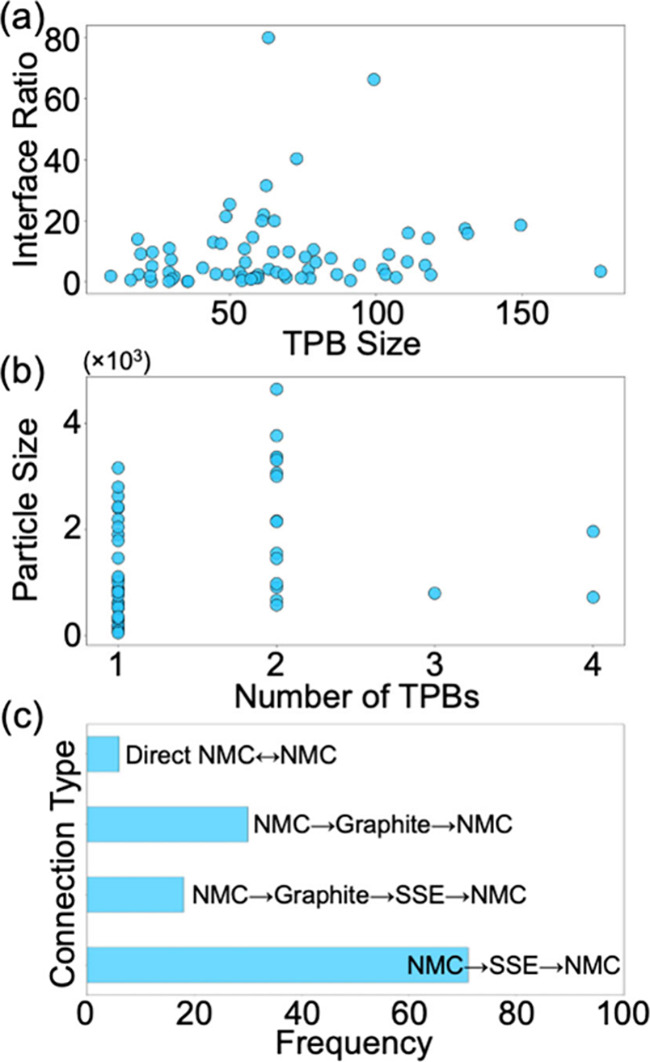
Microstructure morphology analysis. (a) Distribution of TPBs with respect to the TPB size (i.e., the perimeter of NMC–SSE–graphite triangle), and the interface area ratio (i.e., the edge weights for NMC–SSE over NMC–graphite). (b) Distribution of NMC particles with respect to the number of associated TPBs, and the particle size (quantified by pixel count). (c) Bar plot of NMC particle interparticle connections (i.e., the channel to the nearest NMC particle).

We demonstrate the use of microstructure graphs to examine the correlation between the local microstructure morphologies and the local electrochemical states of the individual NMC particles. The local electrochemical states are obtained from energy-resolved three-dimensional full-field TXM (see Supporting Information). Figure 4a–c presents the multimodal imaging example that reflects both the local electrochemical states of NMC particles and the ML-segmented phases, as well as the corresponding graph. It is worth noting that the cathode samples used in this work are discharged, at which a lower Ni oxidation level (represented by lower values of the Ni K-edge energy) indicates a higher degree of completion for the electrochemical reaction. We made two main observations from Figure 4a–c. First, the NMC particle (i.e., particle #1 in Figure 4b) involved in the TPB (labeled by the yellow dashed circle in Figure 4b and the dashed triangle in Figure 4c) has lower Ni oxidation states than the other NMC particles. Second, NMC particle #2 also exhibits a low Ni oxidation state due to its electronic connection with NMC particle #1, given that NMC is also an electron conductor.^39,40^ In addition, Li^+^ can access the surface of particle #2 through the surrounding SSE. These findings corroborate the significant impact of the interparticle connectivity on the electrochemical states of NMC, which will be discussed in greater detail in the next session.

**Figure 4:**
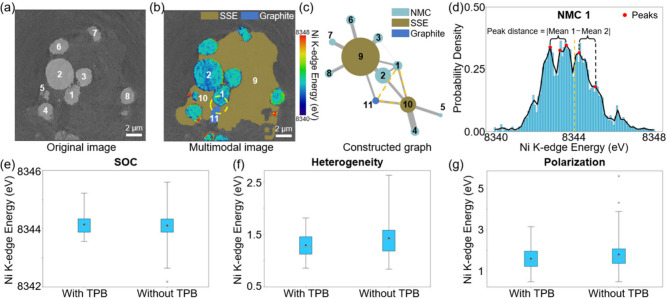
Microstructure–property relationship at the node level. Example of (a) the original image, (b) the corresponding multimodal image, and (c) the constructed graph. (d) The distribution of the electrochemical state (i.e., Ni oxidation state represented by Ni K-edge energy) for #1 NMC in Figure 4a. The distributions of the remaining NMCs are shown in Figure S4. The electrochemical states are described by their distribution means, standard deviations, and peak distances, which are the measurements of intraparticle SOC, heterogeneity, and polarization, respectively. Detected peaks (≥40% of the maximum height) are marked in red dots. Peak distance is calculated as the distance between the mean locations of peaks smaller and larger than 8344 eV. Boxplots of NMC particles with and without TPBs in terms of (e) the SOC, (f) the heterogeneity, and (g) the polarization. The short horizontal orange line represents the median and the small dot shows the mean value. A lower value represents a higher degree of completion for the electrochemical reaction which is desirable.

Figure 4d shows the statistics of the local electrochemical state (represented by the Ni K-edge energy in each pixel) for the #1 NMC particle in the multimodal image, and those of the remaining NMC particles are shown in Supporting Figure S4. The means, standard deviations, and peak distances in these statistical distributions, as indicated in Figure 4d (see details in Supporting Information), correspond to the state of charge (SOC), heterogeneity, and polarization of the NMC particle, respectively. We note that using relative K-edge shifts to evaluate SOC variations is a widely adopted approach in the battery TXM community,^30–32,41^ particularly when comparing samples measured under identical beamline configuration and energy calibration as in this study. We then investigate all the NMC particles in our data set, where 48 out of 350 NMC particles are involved in TPBs, and examine the effect of TPBs involvement on the SOC, heterogeneity, and polarization. As shown in Figure 4e–g, while the averaged SOCs of the NMC particles remain largely independent of their involvement in a TPB, the values of heterogeneity and polarization in NMC particles with TPB are lower than those without TPBs, indicating a more homogeneous and efficient electrochemical reaction. In this regard, our results indicate that the presence or absence of TPB involvement significantly influences the spatial distribution of Ni oxidation states even after significant relaxation. This finding highlights the role of TPB on reaction uniformity as an intrinsic, sustained structural constraint that persists beyond transient polarization effects. Moreover, while the local electrochemical behaviors can also be influenced by other factors including particle size, contact area, local porosity, and proximity to boundary layers, our detailed analyses show that the TPB abundance captures the dominant structural contribution among these correlated factors (see Supporting Information). These results confirm that TPBs promote a spatially more uniform charge–discharge behavior inside an NMC particle and thereby contribute to a prolonged battery lifespan. In particular, the TPB involvement is not considered as a direct, standalone cause of relaxed-state intraparticle heterogeneity, but rather as a key structural factor shaping the surface reaction distribution, which in turn contributes to the development and persistence of internal heterogeneity under solid-state conditions. Furthermore, it is worth noting that the measured Ni oxidation-state distribution reflects the residual heterogeneity after substantial relaxation, rather than the reaction nonuniformity driven by transient polarization under current flow. We believe that the persistence of this heterogeneity after relaxation suggest that significant reaction nonuniformity had developed during cycling, because relaxation is generally expected to reduce the heterogeneity of the Ni oxidation-state distribution.

Microstructure graphs also allow for investigating the microstructure–property relationship at the network level, leveraging established graph analysis tools and theories. Specifically, we quantify key graph-network-level descriptors for NMC particles, including degree, centrality indicators (e.g., closeness, betweenness, and eigenvector), clustering coefficient, and then correlate them with NMC electrochemical states by computing the Pearson correlation coefficient (*r*) (see Supporting Information). A more negative *r* indicates a stronger correlation and a lower Ni K-edge energy (i.e., a more complete electrochemical reaction). Degree quantifies the number of neighbors for a node. Centrality indicators quantify the importance and influence of a node within the overall network, where closeness reflects proximity to all other nodes, betweenness identifies nodes acting as bridges along shortest paths, and eigenvector highlights nodes connected to other influential nodes. Clustering coefficient measures the tendency of a node’s neighbors to form tightly connected groups. As shown in Figure 5a, the heterogeneity and polarization of NMC particles are both strongly correlated with the network descriptors (*r* is large) and have similar correlation patterns, while the correlation between SOC and descriptors is weaker, consistent with the result in Figure 4e. The high absolute *r* values of closeness and eigenvector indicate that the NMC particles are more deeply embedded within the network, which is consistent with their large number of SSE neighbors and has strong correlation with their electrochemical states. In addition, the high weighted degree compared to degree suggests the importance of taking the edge weight (i.e., interface area) into consideration since the interface area affects the efficiency of Li^+^/e^–^ transport. The scatter plots of the top four graph-theoretic metrics with the strongest absolute *r* with the electrochemical states, i.e., intraparticle SOC, heterogeneity, and polarization are shown in Supporting Information, Figures S5–S7.

**Figure 5:**
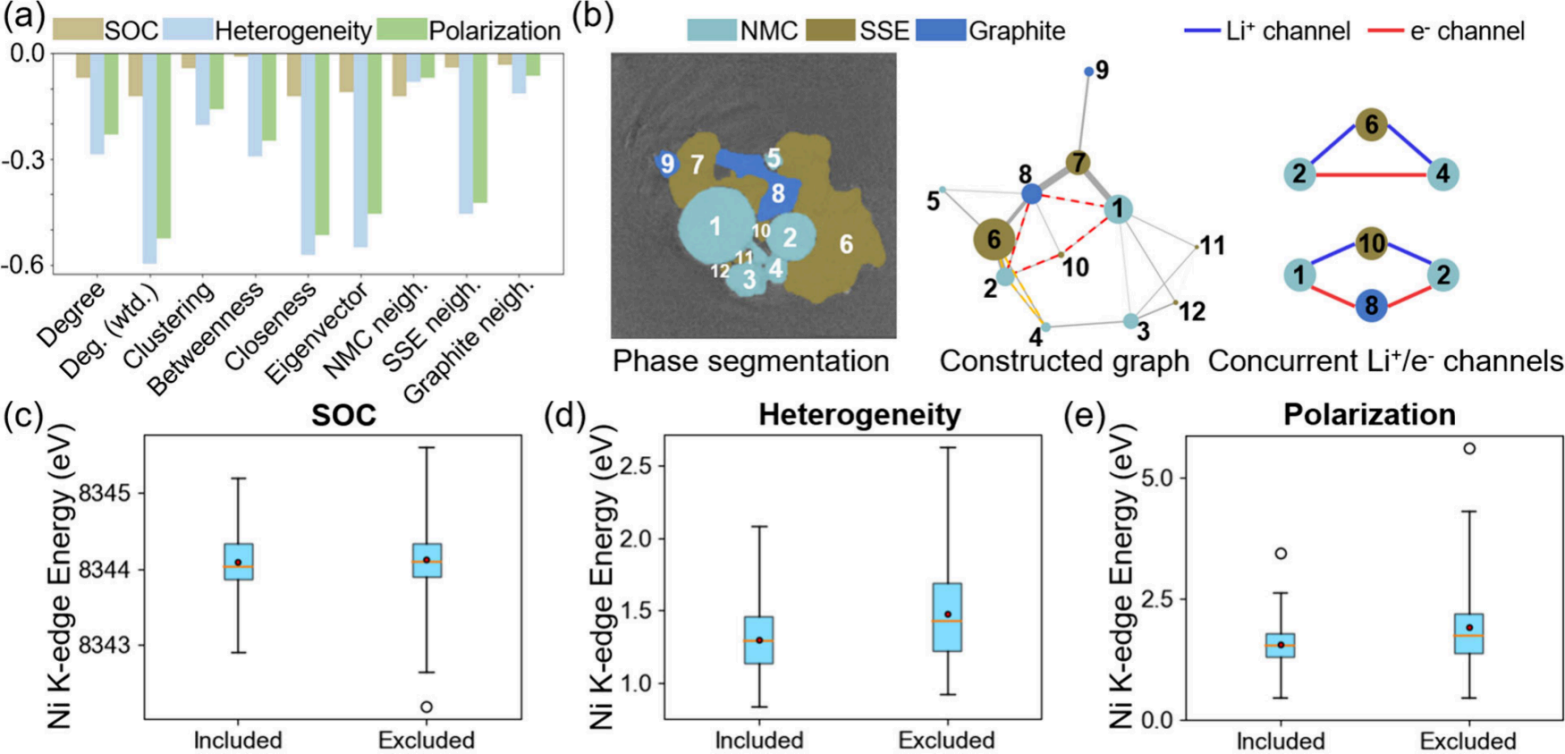
Microstructure–property relationship at the network level. (a) Pearson correlation coefficients between graph-theoretic metrics and electrochemical states (i.e., intraparticle SOC, heterogeneity, and polarization). (b) Demonstration of two types of interparticle connections that entail concurrent Li^+^/e^–^ channels in both the segmented X-ray image and constructed graph. Boxplots of NMC particles included and excluded in the interparticle connections in terms of (c) the intraparticle SOC, (d) heterogeneity, and (e) polarization. The short horizontal orange line represents the median, and the small dot shows the mean value. A lower value represents a higher degree of completion for the electrochemical reaction, which is desirable.

The left panel of Figure 5b shows a segmented microstructure image with NMC particles that have the two types of desirable interparticle connections, which are also highlighted as the dashed lines in the constructed graph (see the middle panel of Figure 5b). The right panel of Figure 5b illustrates these two types of desirable connections between two NMC particles that both can provide concurrent ionic and electronic channels. To further validate this finding, we evaluated the impact of the concurrent Li^+^/e^–^ channels on the electrochemical states (i.e., SOC, homogeneity, and polarization) for a total of 350 NMC particles, among which 136 particles are connected through concurrent Li^+^/e^–^ channels. As shown in Figure 5c–e, the 136 NMC particles with these concurrent channels exhibit reduced heterogeneity and polarization compared to the remaining 214, whereas their SOC shows minor differences. This result unravels the importance of building concurrent Li^+^/e^–^ channels among the NMC particles, in addition to involving them in a TPB.

Importantly, in a recent experiment^38^ (led by a subset of the authors of this work), the graphite/SSE ratios are systematically varied to construct three types of composite cathodes with Li^+^-channel-deficient (LCD), e^–^-channel-deficient (ECD), and balanced Li^+^/e^–^ (BLE) configurations. Among them, the BLE configuration, which inherently corresponds to a higher abundance of effective TPBs, exhibited an optimized capacity and cycling stability. In contrast, ECD electrodes showed reduced capacity and shortened lifetime, while LCD electrodes failed to operate properly due to insufficient ionic percolation. In the present study, we employ ML-enabled graph analysis to quantitatively map the TPB distribution and concurrent transport channels. The results align with the performance trends reported in ref^38^ and support the proposed interpretation that the abundance of TPBs and concurrent Li^+^/e^–^ channels could influence the performance of SSBs.

Automated construction of microstructure graphs from original multimodal microstructure images also enables the application of GNN as a surrogate model to predict the local microstructure–property relationship. Figure 6 demonstrates the prediction of the electrochemical states in each NMC particle via GNN (see Supporting Information). The prediction performances for the intraparticle heterogeneity and polarization are better than SOC, which are consistent with the previous graph-based analyses showing no significant association between SOC and the TPB (Figure 4e) or the concurrent Li^+^/e^–^ channels (Figure 5c). The overall prediction results of these three descriptors indicate a limited predictive capability, which can be attributed to several factors. These factors may include the small data set size, the noise in experimental data, the potential loss of information resulting from the use of two-dimensional (2D) slices instead of three-dimensional (3D) images (specifically, NMC particles that appear to lack TPBs in a 2D slice may in fact possess TPBs in a 3D image), and the relatively small representative microstructure volume (a battery cell contains thousands of NMC particles which significantly exceeds the volume sampled in the present experiment). Although the roles of these factors can only be quantified when larger-scale 3D microstructure data with improved phase contrast become available (acquiring those data would require more advanced tomography techniques), the demonstration in the present work successfully shows the potential of leveraging graph representation and graph learning to directly perform node-level prediction tasks on the experimental data, setting the foundation for the graph-based microstructure inverse design.

**Figure 6:**
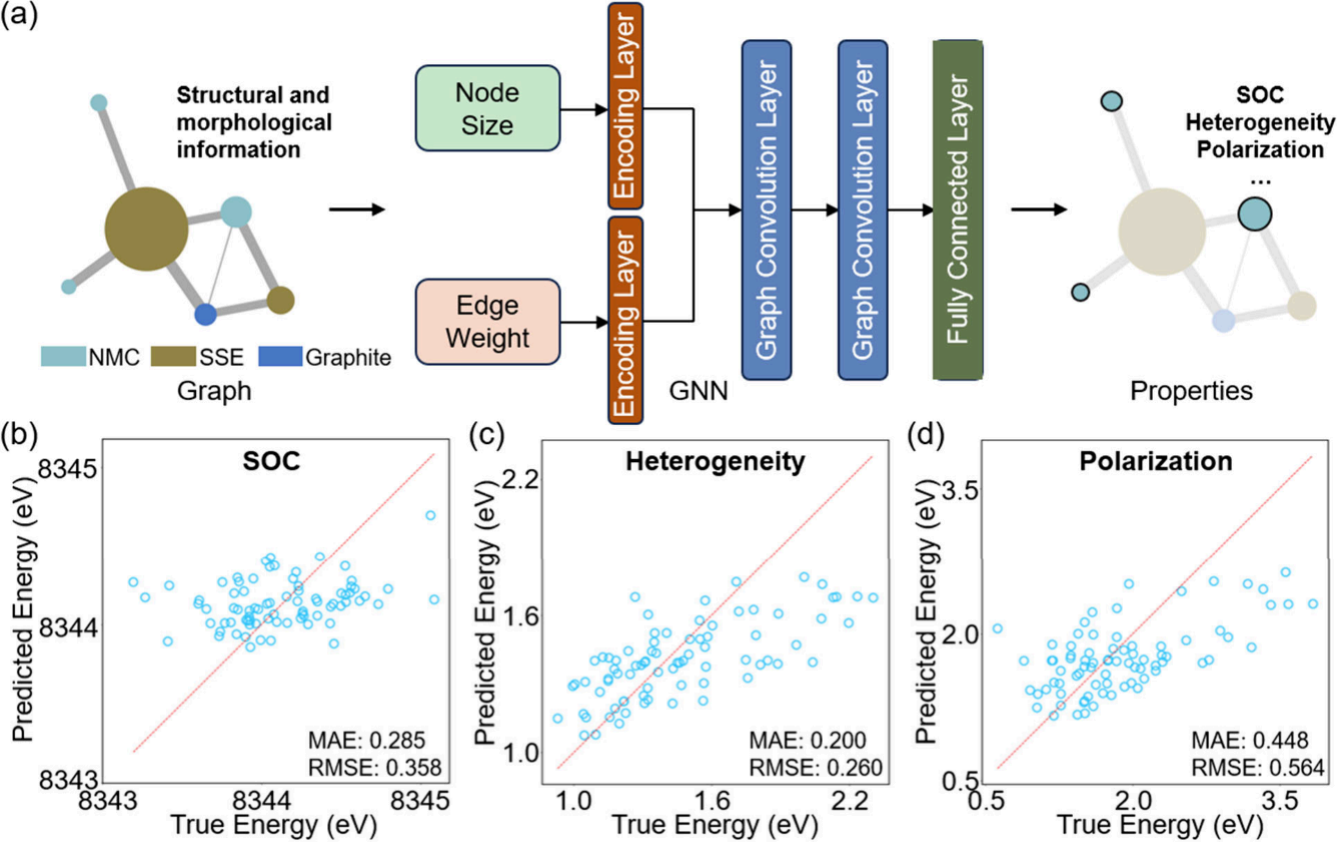
Predicting local microstructure–property relationship based on graphs. (a) The demonstration of predicting NMC electrochemical states using GNN, where the input of the GNN is the structural and morphological information on the graph (i.e., node size and edge weight) and the output is the NMC electrochemical state descriptors, (i.e., the intraparticle SOC, heterogeneity, and polarization). (b–d) GNN prediction versus truth on the testing set for the three electrochemical state descriptors, respectively.

In summary, we developed an ML-enabled workflow for realizing a fast and automated construction of graphs from the experimentally measured X-ray microstructure images of multiphase particulate composites (Figure 2). Using the composite cathode of SSBs as an example, we demonstrated microstructure graphs can enable high-throughput microstructure morphology analysis (Figure 3), the study of the microstructure–property relationships at both the node/edge (Figure 4) and the network (Figure 5) level, and the prediction of the local microstructure–property relationship via GNN (Figure 6). More specifically, based on the automatically constructed microstructure graphs, we analyzed and directly compared the electrochemical states of the NMC particles with and without the concurrent Li^+^/electron channels. The results (Figures 4, 5, and S5–S8) provide much-needed microscopic evidence to the importance of TPBs and coupled ionic/electronic channels using ML-enabled graph analysis of the experimentally measured multimodal images. Furthermore, the statistical tests (i.e., Welch’s two-sample *t*-test and two-way ANOVA) support our findings (see Supporting Information). This particle-resolved approach provides evidence for the correlation between structural features, i.e., the triple phase boundaries and the concurrent Li^+^/e^–^ channels, and the residual electrochemical heterogeneity observed in the relaxed state. These findings suggest that such structural configurations may facilitate electrochemical reactions by mitigating local transport resistances for both Li^+^ and e^–^, thereby contributing to a more uniform distribution of overpotential and current density during operations.^42,43^ Thus, our results emphasize not only the importance of selecting appropriate particle morphologies but also the need to control particle self-assembly during electrode fabrication of SSBs. For example, it has been shown that tuning the particle size ratio between the active material and the solid electrolyte can potentially regulate the TPB density and network connectivity.^44^ However, establishing more quantitative correlation between the processing parameters and the microstructures of composite cathode represents an open challenge in the field of SSBs. The graph-based microstructure representation demonstrated in this work can potentially function as a critical enabler for the inverse process design, i.e., searching for the process parameters that lead to the desirable microstructures. For example, the graph-theoretic metrics can be used to construct a topology-aware loss function in an active-learning-based search for the process parameters.

We also outline several other challenges and opportunities that are specifically related to solid-state batteries. First, multimodal X-ray imaging in the present work has an ultrahigh spatial resolution of 20 nm, which allows for the accurate imaging of the local geometry and topology. Yet, this high resolution comes at the expense of probing a relatively small fraction of the entire composite battery cathode, which may not necessarily be sufficient to serve as a representative volume element (RVE) for the entire cathode. For example, a recent study suggests that a representative volume containing 100 to 1,000 particles corresponds to the scale of heterogeneity in the battery composite electrode,^45^ which is significantly larger than the number of NMC particles herein. Second, the imaging modality used in the present work, which is different from that in ref 45, leads to relatively low image contrast among the background, SSE, and graphite, as shown in Figure S1. This low contrast makes it exceedingly challenging for human researchers to perform 3D annotation as the ground truth training data of a ML-based segmentation model. Addressing these two challenges will need more experiments and/or coordinated data sharing, as well as the combined use of additional imaging modalities. The present ML-enabled graphic analysis, despite being focused on 2D slices and relatively small microstructure volume, provides a statistically rigorous, self-consistent evaluation of the local microstructure–property relationship at both the individual particle level and the particle network level. Our ML-enabled graph analysis tools can be readily extended to analyze larger-scale (i.e., practically sized) multimodal 3D microstructure data sets once they become available, and the high scalability of the graph-based approach makes it particularly efficient and suitable for harnessing large-scale microstructure data. For example, an earlier study demonstrates a strong scalability of GNN training to thousands of nodes even on a single graphics processing unit (GPU) node.^19^

More broadly, our results demonstrate the potential of leveraging graphs to achieve data-driven forward prediction of the microstructure–property relationship and the inverse process design in multiphase particulate composites, where one key challenge has been finding an informative low-dimensional representation of the high-dimensional microstructure data. Specifically, graph-based microstructure representation uniquely allows for encoding both the morphological and topological information on the microstructure as well as phase-dependent local properties (e.g., conductivities) as node/edge features. In the forward prediction, these unique capabilities can potentially lead to an improved prediction accuracy over several baseline ML models, as has previously been demonstrated in polycrystalline microstructures.^19^ In the inverse process design, as briefly discussed above, the graph-theoretic metrics can be directly used to construct a topology-aware loss function in active learning that goes beyond the use of simple geometric features, such as the mean particle size and size distribution. Overall, we expect that the results in this work would stimulate more efforts of constructing and harnessing graphs to revolutionize the design and discovery of a wide range of multiphase particulate composite materials.

## Supplementary Material



## Notes

Details of solid-state battery fabrication and characterization; Graph construction enabled by ML-based automated phase segmentation; Electrochemical state quantification for NMC particles; Graph-theoretic metrics; GNN for predicting NMC electrochemical states; Statistical tests for results presented in Figures 4e–g and 5c–e; Two-way ANOVA analysis for heterogeneity and polarization; Analyses of the potential confounders (particle size, contact area, local porosity, and proximity to boundary layers) (PDF)
