# Supplementary material for: Machine Learning Enabled Graph Analysis of Particulate Composites: Application to Solid-State Battery Cathodes
Source: ACS Energy Lett. 2026 May 14;11(6):4334–42. doi: 10.1021/acsenergylett.5c04258 (PMC13270636; doi:10.1021/acsenergylett.5c04258)
Supplement: Supplementary file 1 [file nz5c04258_si_001.pdf]

Supporting Information

Machine Learning Enabled Graph Analysis of Particulate Composites: Application to Solid-state Battery Cathodes

Zebin Li[1], Shimao Deng[2], Yijin Liu[2*], Jia-Mian Hu[1*]

[1]*Department of Materials Science and Engineering, University of Wisconsin-Madison, Madison, WI 53706, USA*

[2]*Walker Department of Mechanical Engineering, University of Texas at Austin, Austin, TX 78712, USA*

[*]E-mails: liuyijin@utexas.edu (Y.L.) or jhu238@wisc.edu (J.-M.H.)

Solid-state battery fabrication and characterization

Materials and cell assembly. $LiNi_{0.8}Co_{0.1}Mn_{0.1}O_2$ (NMC811) powder coated with $LiNbO_3$ (NEI Corporation), along with Li_6PS_5Cl (LPSCl) (NEI Corporation), and lithium metal are employed as the cathode active material, solid-state electrolyte (SSE), and anode, respectively. The composite cathode is formulated by thoroughly mixing NMC811, LPSCl, and graphite in a mass ratio of 6:4:2. For cell fabrication, approximately 150 mg of wet ball-milled LPSCl powder[1] is pressed at 530 MPa for 1 minute using a polyether ether ketone (PEEK) mold to form the SSE layer. Subsequently, around 40 mg of the composite cathode mixture is carefully placed onto the SSE layer and compacted at 867 MPa for 2 minutes. A lithium metal foil, approximately 100 μm thick, is then positioned on the opposite side of the SSE without additional compression. The entire assembly is enclosed in a custom-designed cell casing and tightened to 9 MPa to ensure intimate interfacial contact for electrochemical evaluation.

Galvanostatic charge–discharge testing is conducted using a Land CT2001A battery testing system. The cells are initially cycled at a rate of 0.1 C for the first two formation cycles, after which the current rate is increased to 0.33 C for subsequent cycles. All electrochemical measurements are performed within a voltage range of 2.8 to 4.3 V versus Li/Li^+.

Full-field X-ray imaging. Three-dimensional full-field transmission X-ray microscopy (TXM) is performed at the full-field X-ray imaging (FXI) beamline (18-ID) of the National Synchrotron Light Source II (NSLS-II), Brookhaven National Laboratory. We clarify that all Ni K-edge TXM measurements were performed ex situ. The time interval between cell preparation and measurement is constrained by beamtime logistics and experimental scheduling, and is typically on the order of 1–2 days. The imaging is carried out with a spatial resolution corresponding to a pixel size of 20 nm. Regarding sample preparation, cathode particles are gently detached from the electrode surface within an argon-filled glove box and immediately loaded into capillary tubes. These are subsequently sealed with epoxy inside the glove box to prevent air exposure. Tomographic projection images are acquired over a 0–180° rotation range under fly scan mode. For the three-dimensional X-ray absorption near-edge structure (XANES) analysis, the incident X-ray energy is scanned across the Ni K-edge, from 8210 eV to 8700 eV, using 63 discrete

energy steps. TXM data reconstruction and analysis are performed using the TXM-Wizard software package[2].

Graph construction enabled by ML-based automated phase segmentation

Phase segmentation by U-Net. U-Net is a commonly used architecture build upon convolution neural network for image segmentation proposed in 2015[3]. It is comprised of a symmetric encoder-decoder structure, where the skip connections directly link corresponding layers between the encoder and decoder paths. Such a design of the skip connection enables the U-Net to capture information at different levels, improving its capacity in segmenting complex patterns. Specifically, the encoder is responsible for reducing the spatial dimensions and extracting features (i.e., downsampling) at different scales for the input image, while the decoder then restores the extracted feature map (combined with the features from the corresponding encoder through skip connection) to the original dimension via transposed convolutional operation (i.e., upsampling). The final output of the U-Net is the segmentation map of the input image.

In our work, we develop a customized U-Net enhanced by a dual attention mechanism using PyTorch[4]. The dual attention blocks are incorporated into the decoder skip connections to refine feature fusion by jointly applying class-wise attention, which emphasizes features relevant to semantic class discrimination, and instance-wise attention, which enforces separation between neighboring instances. This design allows the network to suppress irrelevant background activations while enhancing features that contribute to both accurate class assignment and boundary delineation. The overall architecture contains three levels in both the encoder and decoder. Each encoder block consists of two convolutional layers with ReLU activation and dropout, followed by max-pooling for downsampling. The decoder mirrors this structure with transposed convolutions for upsampling, attention-modulated skip connections, and convolutional layers to refine the merged features. The final segmentation map is produced by a 1×1 convolutional layer that projects the decoded features onto the target number of classes.

For training, we employ a combined loss function that integrates the Dice loss with a contour-aware binary cross-entropy loss, thereby balancing region consistency with precise boundary localization. Optimization is performed with the AdamW optimizer (learning rate 1×10^{-4}, weight decay 1×10^{-4}). The dataset (100 images in total) is partitioned into training, validation, and testing with the ratio of 70%, 15%, and 15%. To enhance robustness and generalization, data augmentation is applied to the training set, including random horizontal flips, small rotations, and color jittering. Model performance is evaluated using the mean Intersection-over-Union (mIoU) across all classes (background, SSE, NMC, and graphite), along with class-specific IoU values.

Post-processing of phase segmentation by watershed algorithm. The watershed algorithm is a classical image processing technique based on topographic analysis and is particularly effective for separating touching or overlapping objects in images[5]. It treats the image (usually a grayscale image) as a topographic surface, where valleys and peaks represent low-intensity and high-intensity areas, respectively. Then, a flooding process is simulated over the topographic surface (i.e., image), in which water gradually fills the basins from predefined markers. The predefined markers are the starting points of the flooding process, and they correspond to different objects or areas in the image. In particular, a distance map is first calculated, where each pixel within the object is assigned a value representing its distance to the nearest background pixel. The center pixel within the object has the highest distance value (i.e., peak), therefore treated as the predefined marker. As the flooding process from different markers expands, the segmentation map

(i.e., boundaries between objects) forms when different basins merge, resulting in the separation of individual objects.

Furthermore, a convexity-based iterative refinement is simultaneously implemented to further enhance the performance of post-processing. Specifically, after each segmentation iteration, the convexity of every separated object (i.e., NMCs) is evaluated. Objects with convexity below a predefined threshold (set to 0.95) are considered potentially under-segmented and are subjected to further watershed processing. Such a strategy allows separating the majority of the connected NMC particles, for example, those shown in **Fig. 2**. For NMC particles with complex, irregular shape or severe agglomeration, occasional over-segmentation or residual merging still occurs. This challenge could be potentially mitigated by adopting more sophisticated learning-based segmentation methods[6] in which case large number of images labeled by domain expert may be needed for the model training.

Graph construction. The instance segmentation maps obtained after the post-processing of phase segmentation maps from X-ray images (they are 2D virtual slices, same thereafter) are converted to graph networks, in which the geometric information (e.g., connections among different phases, particle sizes, etc.) of the microstructures for SSBs can be represented in an efficient way. Specifically, each object (e.g., particles of NMC, SSE, and graphite) in the instance segmentation map is considered as a node in the graph, and the areas of the masks for objects are considered as the particles' sizes. Besides, the edges among different nodes represent their adjacency relationships. During segmentation, two objects are typically not allowed to truly overlap (i.e., the same pixel cannot belong to two instances simultaneously). Therefore, the adjacency between two objects is determined using a morphological dilation-based proximity criterion. Specifically, for each object, a binary mask is dilated with a 7×7 square structuring element (i.e., a flat kernel of ones), which expands the object by up to three pixels in all directions under the Chebyshev distance[7]. Two objects are considered adjacent if the dilated mask of one object overlaps with the binary mask of the other. The degree of overlap is quantified as the number of intersecting pixels between the dilated region of the first object and the original region of the second object. This pixel count reflects the extent of near-boundary contact rather than true geometric overlap, since instance masks are mutually exclusive. An undirected edge is added to the graph when this count is nonzero, and the edge weight is defined as a scaled version of this intersection value, thereby encoding the strength of spatial proximity between objects.

Electrochemical state quantification for NMC particles

The electrochemical state of NMC particles refers to the Ni oxidation distribution (represented by the Ni K-edge energy distribution) and it is represented by the pixel intensity distribution in the TXM image. By applying the instance segmentation map on the X-ray image, it allows the identification of each NMC particle. Then, the statistics (i.e., mean, standard deviation, and peak distance) of the pixel intensity distribution for each NMC particle are extracted to provide a concise description for its electrochemical state. The pixel distribution is cut at 8340 and 8348 eV according to the Ni adsorption edge. To calculate the peak distance, the pixel histogram of each particle is first smoothed and then analyzed using the *find_peaks* function in Scipy to identify dominant peaks, where a peak is considered dominant if its height is at least 40% of the maximum peak height. If exactly two dominant peaks are present, their separation directly defines the distance; for distributions with multiple peaks, the average positions of the peaks on either side of the reference energy (8344 eV) are compared. It is worth noting that relating the observed Ni K-edge shifts to an approximate change in Li content (Δx) or state of charge (ΔSOC) would provide a more intuitive sense of the physical and technological significance of the reported differences.

However, such quantification would require an appropriate calibration framework, for example using reference spectra with known Ni valence states and linear-combination analysis, which is beyond the scope of the present dataset. The aim of this study is therefore not to assign absolute Δx or ΔSOC values from the measured edge shifts, but to use the relative Ni K-edge shifts under identical measurement conditions as an internal indicator of spatial differences in reaction extent.

Graph-theoretic metrics

We characterize the connectivity of NMC particles in the graphs using several standard graph-theoretic metrics[8]. The degree of a node quantifies how many neighboring particles it is directly connected to, and the weighted degree extends this by summing the contact strengths along all its edges. The clustering coefficient measures the ratio of the number of triangles in the constructed graph to the total number of geometrically allowable triangles, reflecting how densely interconnected the local environment is. Betweenness centrality evaluates how frequently a node lies on the shortest paths connecting other pairs of nodes, thus indicating whether it acts as a bridge for transport across the network. Closeness centrality measures how close a node is to all other nodes on average, based on shortest-path distances, and captures how efficiently a particle can interact with the entire system. Eigenvector centrality assigns higher importance to nodes that are connected to other well-connected nodes, identifying particles embedded in influential regions of the graph. Moreover, we also count the number of neighbors of each type, i.e., the number of NMC, SSE, and graphite neighbors, around each NMC particle to quantify the local chemical environment.

GNN for predicting NMC electrochemical states

Graph neural networks (GNNs) provide a natural framework for analyzing microstructures, as they can capture both node-level attributes (e.g., particle size) and structural information (e.g., inter-particle connections). In our setting, the SSB cathode microstructure is represented as a heterogeneous graph where NMC particles, SSE and graphite phases are nodes of different types, and edges denote physical contacts. By extending message passing to heterogeneous graphs, GNNs are able to learn predictive mappings from microstructural connectivity and attributes to electrochemical states.

We implement a heterogeneous node regression model based on two stacked NNConv layers within a HeteroConv framework, combined with GELU activation, dropout, and LayerNorm, using PyTorch Geometric[9]. The edge attribute (i.e., physical contact strength) is encoded through a small neural network before aggregation, and the residual connection is applied to stabilize training. The target task is to predict NMC electrochemical state descriptors, i.e., intra-particle SOC, heterogeneity, and polarization (they are the means, standard deviations, and peak distances of electrochemical state distributions) at the node level. Data preprocessing, i.e., z-score standardization is performed to improve the training stability. The dataset (73 images in total) is split into training, validation, and testing with the ratio of 60%, 20%, and 20%. The AdamW optimizer is used with the learning rate of 1×10^{-3} and the weight decay of 1×10^{-2}), gradient clipping (norm ≤ 2), and an early stopping patience of 50 epochs. The mean squared error (MSE) loss is adopted for the model training. Root mean squared error (RMSE) and mean absolute error (MAE) are used to evaluate the prediction performance.

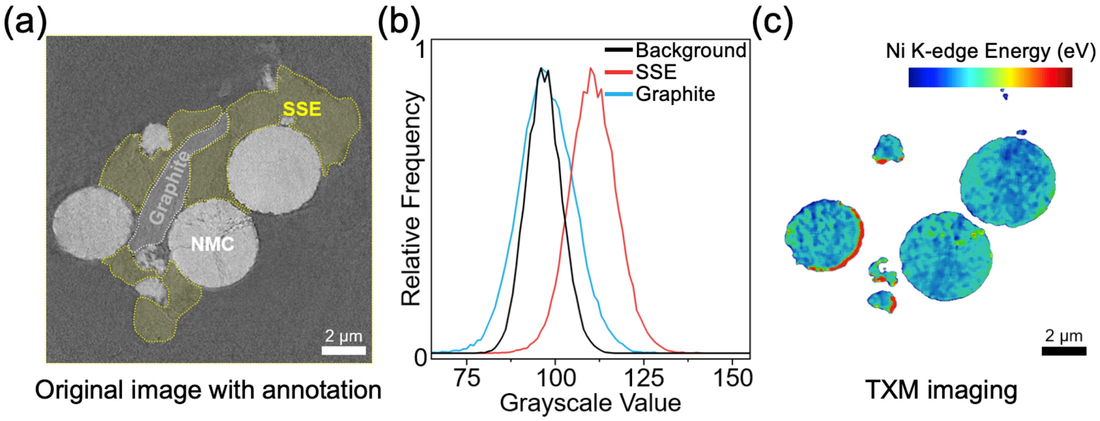

Figure S1. (a) Original X-ray image with expert annotation of NMC, SSE, and graphite. **(b)** The pixel intensity distributions of the SSE, the graphite, and the background in (a), reveal their similarity. **(c)** Example of local electrochemical states (i.e., Ni oxidation states represented by the Ni K-edge energy) of NMC particles obtained by TXM imaging for (a).

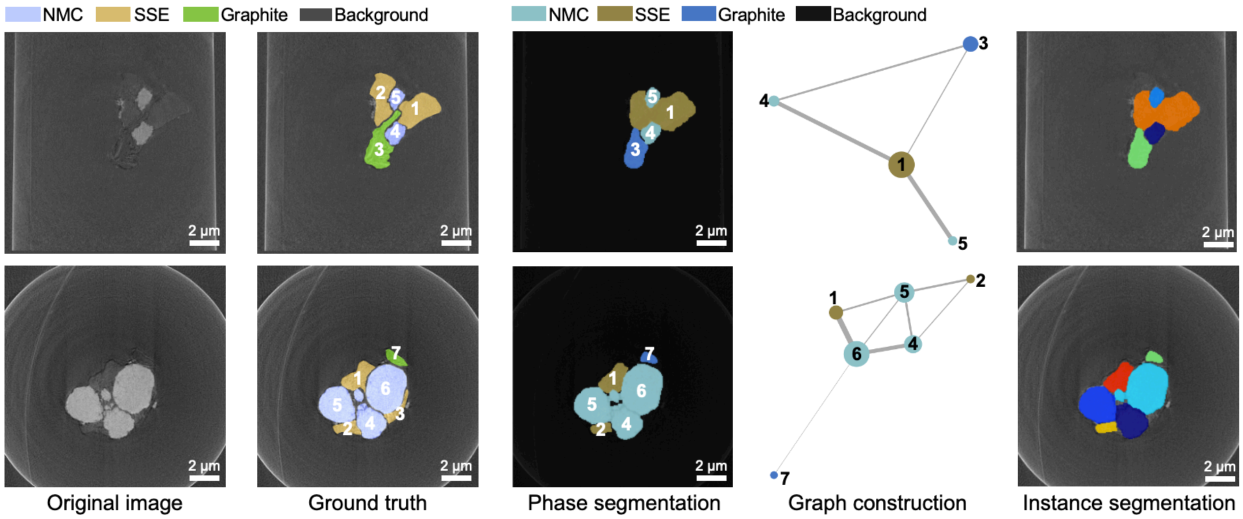

Figure S2. Example results of graph construction enabled by ML-based automated phase segmentation.

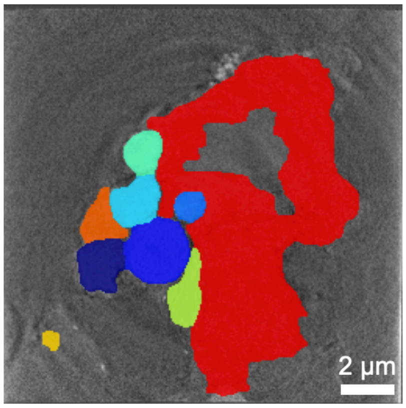

Figure S3. Example of the instance segmentation result after performing the watershed algorithm. Each object in the X-ray image is represented by a unique color.

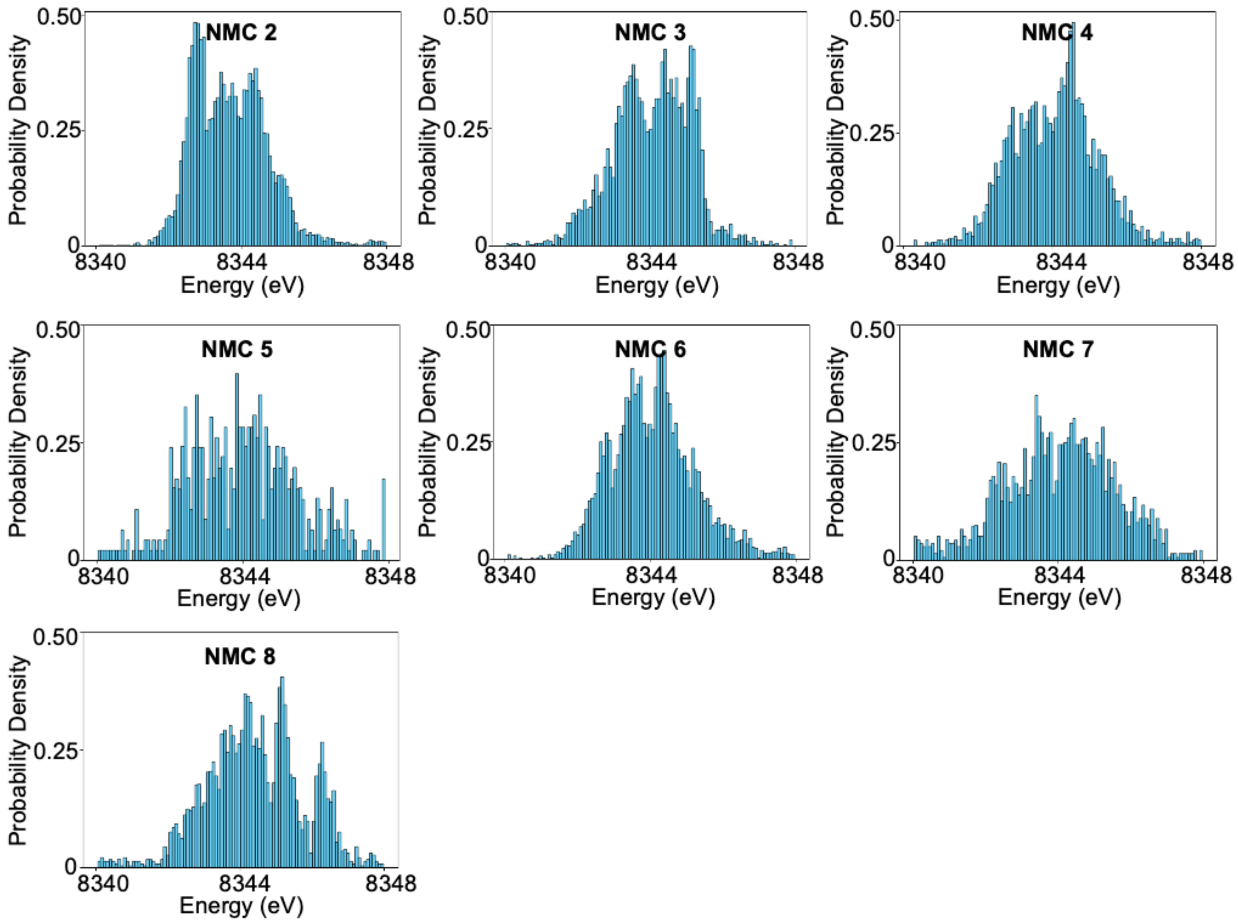

Figure S4. The electrochemical state (i.e., Ni oxidation state, represented by the Ni K-edge energy) distributions of NMC particles #2 – #8 in **Fig. 4a**.

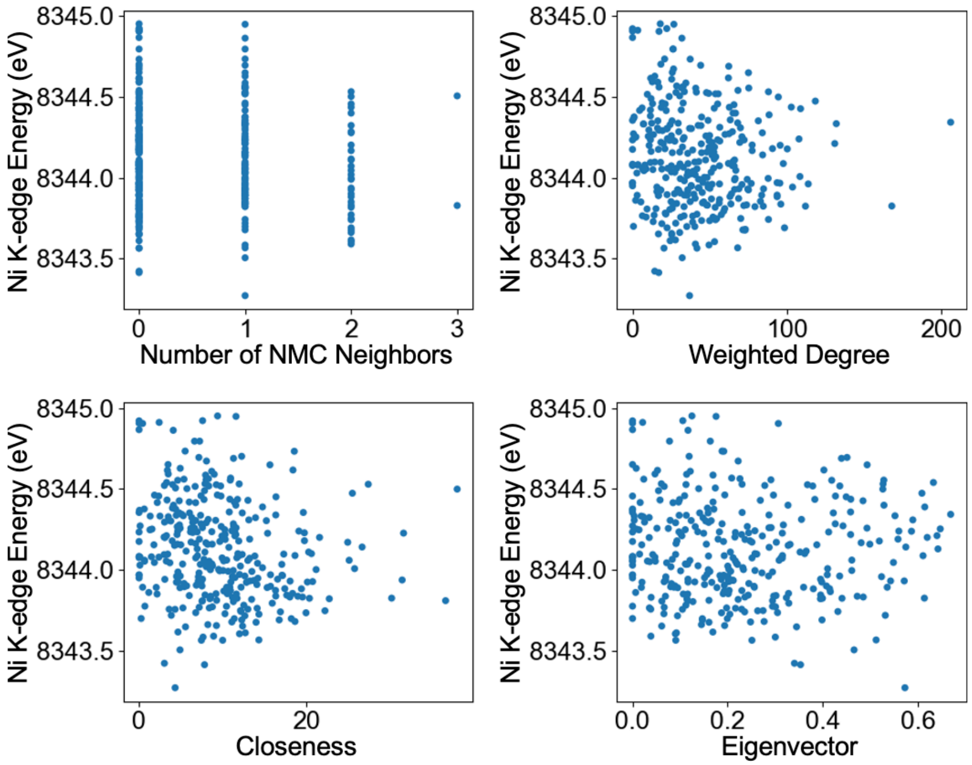

Figure S5. Scatter plots of the top four graph-theoretic metrics with the strongest absolute Pearson correlations with SOC.

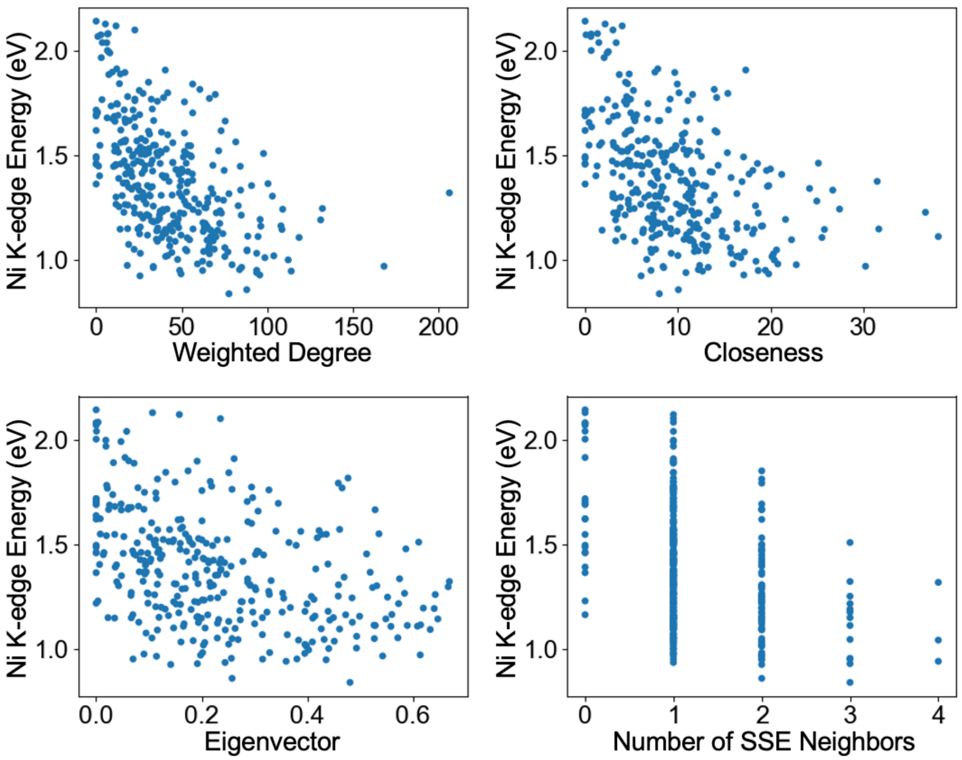

Figure S6. Scatter plots of the top four graph-theoretic metrics with the strongest absolute Pearson correlations with heterogeneity.

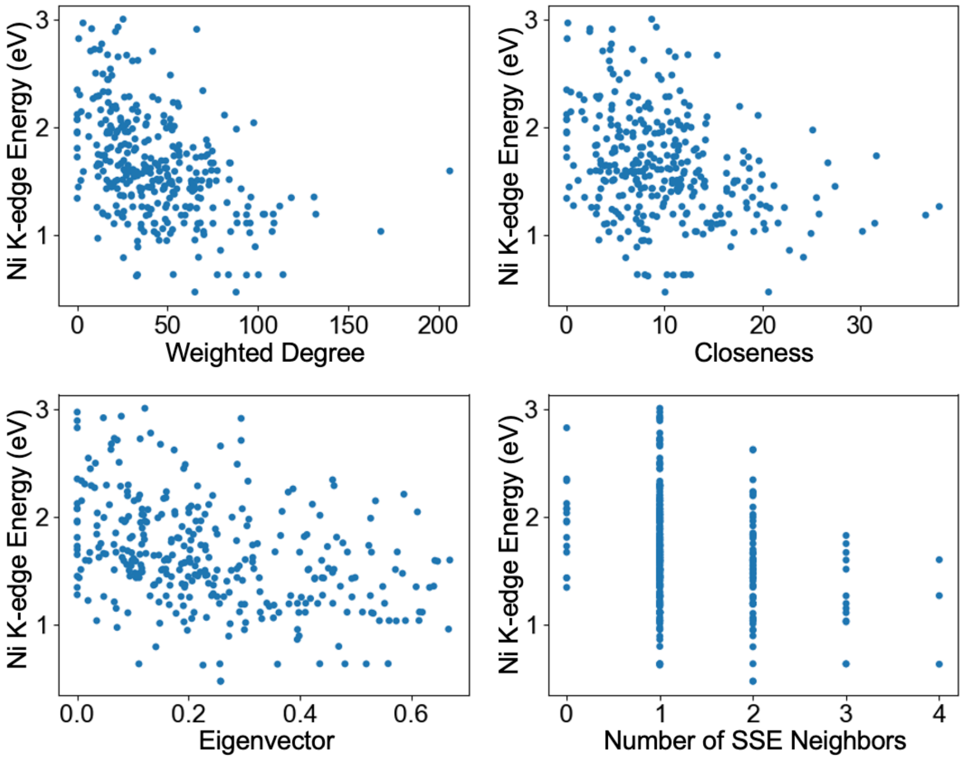

Figure S7. Scatter plots of the top four graph-theoretic metrics with the strongest absolute Pearson correlations with polarization.

Statistical tests for results presenting in Figs. 4e-g and Figs. 5c-e

We employ Welch's two-sample *t*-test with a significance level of α = 0.05 on the data presented in Figs. 4e-g and Figs. 5c-e, since it does not assume equal variances and provides a robust inference under unequal sample sizes, which fits our cases[10]. The results are shown in Table S1. The effect sizes are evaluated by Cohen's *d* measurement[11]. From Table S1, it can be observed that the statistical tests support our conclusion that the TPB and the concurrent existence of Li^+ and e^- pathways have positive impact on the heterogeneity (Fig. 4f and Fig. 5d) and polarization (Fig. 4g and Fig. 5e). Although the results for SOC (Fig. 4e and Fig. 5c) are not statistically significant, they are consistent with our findings in the manuscript that the averaged SOCs of the NMC particles remain largely independent of their involvement in a TPB or concurrent existence of Li^+ and e^- pathways.

Table S1. Statistical Test Results for Figs. 4e-g and Figs. 5c-e

Figures	P-Values	Effect Sizes	Confidence Intervals
Fig.4e	0.542	0.08	[-0.075, 0.144]
Fig.4f	0.00165	-0.43	[-0.212, -0.053]
Fig.4g	0.028	-0.29	[-0.377, -0.025]
Fig.5c	0.346	-0.10	[-0.125, 0.044]
Fig.5d	2.25×10^{-8}	-0.58	[-0.233, -0.114]
Fig.5e	1.17×10^{-7}	-0.53	[-0.486, -0.228]

Two-way ANOVA analysis for heterogeneity and polarization

We further conduct a two-way Analysis of Variance (ANOVA) to investigate the effects of TPB involvement (present vs absent) and concurrent Li^+ and e^- transport pathways (present vs absent) on heterogeneity and polarization. We analyze heterogeneity and polarization separately and do not investigate SOC due to its independence of TPB and concurrent Li^+/e^- pathways involvement. To address the non-normality and heteroscedasticity commonly observed in electrochemical characterization data, we apply a log-transformation to the heterogeneity and the polarization data (i.e., the standard deviation and the peak distance of the electrochemical state represented by the Ni K-edge energy distribution). The two-way ANOVA results for polarization and heterogeneity are shown in Table S2 and S3.

Table S2. Two-way ANOVA Results for Heterogeneity

	Sum of Squares	Degrees of freedom	F-statistic	P-value
With TPB	0.073097	1	5.101139	0.024532
With concurrent Li^+/e^- pathways	0.347206	1	24.230103	0.000001
Interaction of TPB/concurrent pathways	0.001998	1	0.139463	0.709044
Residual	4.958020	346	NaN	NaN

Table S3. Two-way ANOVA Results for Polarization

	Sum of Squares	Degrees of freedom	F-statistic	P-value
With TPB	0.096885	1	1.923031	0.166416
With concurrent Li^+/e^- pathways	0.979693	1	19.445506	0.000014
Interaction of TPB/concurrent pathways	0.003176	1	0.063037	0.801908
Residual	17.431979	346	NaN	NaN

These results suggest that TPB involvement and concurrent Li^+/e^- pathways have distinct contributions to electrochemical behavior. After accounting for concurrent pathway accessibility, TPB involvement remains significantly associated with heterogeneity. While for polarization, only concurrent pathway remains significant, the TPB involvement does not show an independent effect. The two-way ANOVA results are mostly consistent with our previous statistical analysis (Table S1 in the Supporting Information). Specifically, TPB involvement is significantly associated with heterogeneity (Fig. 4f), while concurrent pathway accessibility is significantly associated with both heterogeneity and polarization (Fig. 5d–e). For polarization, the marginal significance observed for TPB involvement in the pairwise test ($p = 0.028$ in Fig. 4g) does not persist after accounting

for concurrent pathway accessibility in the two-way ANOVA, indicating that this effect is not independent.

These results suggest that concurrent Li^+/e^- pathway accessibility has a stronger and more consistent effect than TPB involvement across both heterogeneity and polarization. Nevertheless, TPB involvement still plays an important role, particularly as a local structural feature influencing heterogeneity. In addition, we would like to emphasize that the analyzed experimental data are noisy and 2D, which may introduce bias in the quantitative estimates of the effects. The primary aim of the analyses shown in Figs. 4 and 5 is to demonstrate the use of graph representation for downstream tasks, thereby corroborating the effectiveness of graph representation in revealing the local microstructure-property relationship in multiphase particulate composites.

Analyses of the potential confounders (particle size, contact area, local porosity, and proximity to boundary layers)

Particle size. To decouple NMC particle size from TPB abundance, we investigate the correlation between the particle size and the electrochemical state (i.e., SOC, heterogeneity, and polarization) for NMCs involved in one TPB and two TPBs, since most of NMCs involved in TPBs fall into these two categories according to Fig. 3b. The scatter plots are shown in Figure S8. The first row shows results for NMCs involved in one TPB, while the second row for those involved in two TPBs. It can be observed that there is no clear pattern, which indicates that particle size alone does not have significant impact on the electrochemical state, suggesting that the TPB abundance plays a more critical role.

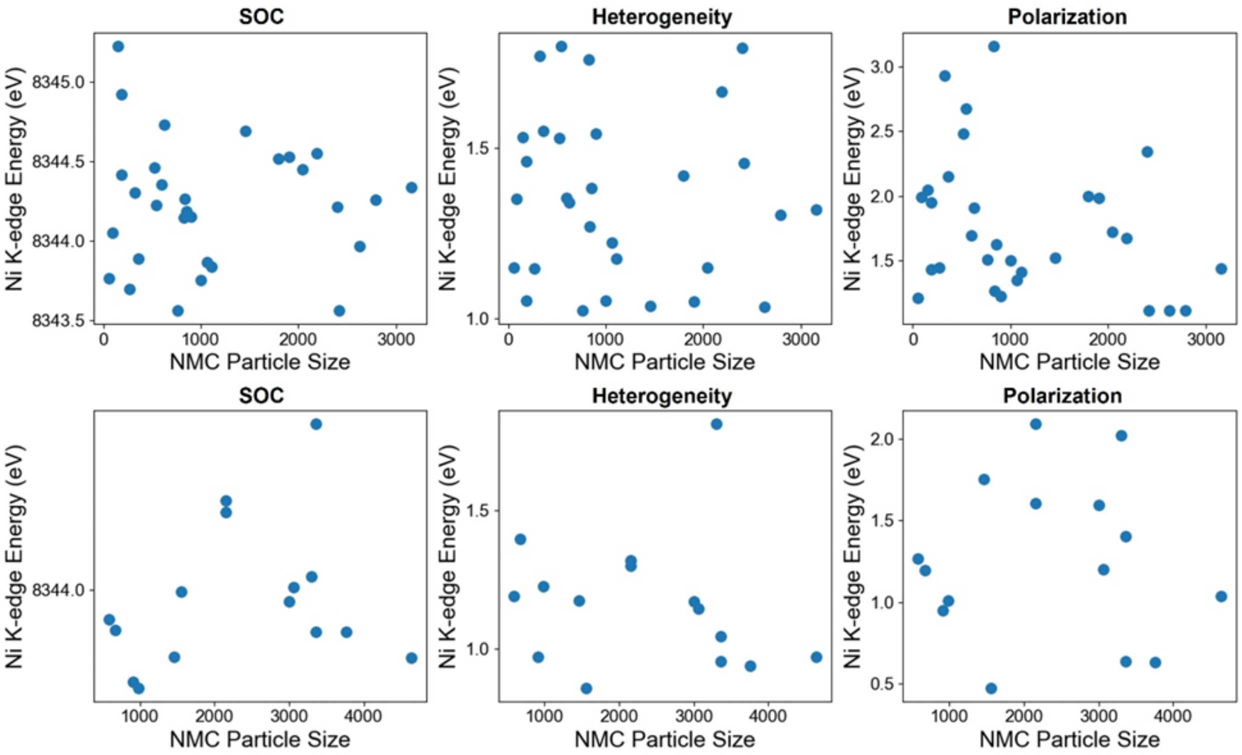

Figure S8. Scatter plots of the electrochemical state (i.e., SOC, heterogeneity, and polarization) with respect to the NMC particles size for NMCs involved in one TPB (the first row) and two TPBs (the second row).

Contact area and local porosity. Contact area and porosity are structurally coupled with TPB formation and ion/electron transport pathways. In our analysis, we explicitly quantify ion and electron channel connectivity at the particle surface, which inherently captures the effect of contact area and local porosity. Therefore, these factors are not independent variables but are embedded within the TPB/channel descriptors used in this study.

Proximity to boundary layers. Proximity to interfaces may influence ionic and electronic conductivity, particularly ionic transport. However, since our analysis is performed on a particle-resolved basis within the same electrode environment, particles with and without TPBs are subject

to comparable boundary conditions. Thus, while boundary effects may contribute to local variations, they do not systematically bias the TPB-based comparisons.

In summary, though these structural parameters may contribute to local heterogeneity, TPB abundance captures the dominant structural contribution among these correlated descriptors.

Supporting References

1. Wang, Y.; Hao, H.; Naik, K. G.; Vishnugopi, B. S.; Fincher, C. D.; Yan, Q.; Raj, V.; Celio, H.; Yang, G.; Fang, H.; Chiang, Y. M.; Perras, F. A.; Jena, P.; Watt, J.; Mukherjee, P. P.; Mitlin, D. Mechanical milling–induced microstructure changes in argyrodite LPSCl solid-state electrolyte critically affect electrochemical stability. *Advanced Energy Materials* **2024**, *14* (23), 2304530.

2. Liu, Y.; Meirer, F.; Williams, P. A.; Wang, J.; Andrews, J. C.; Pianetta, P. TXM-Wizard: a program for advanced data collection and evaluation in full-field transmission X-ray microscopy. *Journal of Synchrotron Radiation* **2012**, *19* (2), 281–287.

3. Ronneberger, O.; Fischer, P.; Brox, T. In U-net: convolutional networks for biomedical image segmentation, *International Conference on Medical image computing and computer-assisted intervention*, Springer: **2015**; pp 234–241.

4. Paszke, A.; Gross, S.; Massa, F.; Lerer, A.; Bradbury, J.; Chanan, G.; Killeen, T.; Lin, Z.; Gimelshein, N.; Antiga, L., Pytorch: An imperative style, high-performance deep learning library. *Advances in neural information processing systems* **2019**, *32*.

5. Vincent, L.; Soille, P., Watersheds in digital spaces: an efficient algorithm based on immersion simulations. *IEEE Transactions on Pattern Analysis & Machine Intelligence* **1991**, *13* (06), 583–598.

6. Kirillov, A.; Mintun, E.; Ravi, N.; Mao, H.; Rolland, C.; Gustafson, L.; Xiao, T.; Whitehead, S.; Berg, A. C.; Lo, W.-Y. In Segment anything, *Proceedings of the IEEE/CVF International Conference on Computer Vision*, **2023**; pp 4015–4026.

7. Soille, P., Morphological image analysis: principles and applications. Springer: **1999**; Vol. 2, No. 3.

8. Latora, V.; Nicosia, V.; Russo, G., Complex networks: principles, methods and applications. Cambridge University Press: **2017**.

9. Fey, M.; Lenssen, J. E., Fast graph representation learning with PyTorch Geometric. *arXiv:1903.02428 2019*.

10. Ruxton, Graeme D. "The unequal variance t-test is an underused alternative to Student's t-test and the Mann–Whitney U test." *Behavioral ecology* **2006** *17*(4), 688-690.

11. Cohen, Jacob. Statistical power analysis for the behavioral sciences. routledge, 2013.
